# Perceptions and Attitudes on Medical Education Extended Reality Applications from a Single Anesthesiology Residency Program

**DOI:** 10.1089/jmxr.2024.0015

**Published:** 2024-07-24

**Authors:** Daniel Cho, Alexandra Berman, John Edwin Rubin, Rohan Jotwani

**Affiliations:** 1 Department of Anesthesiology, NewYork Presbyterian Hospital/Weill Cornell Medical Center, New York, NY, USA.; 2 Department of Anesthesiology, Weill Cornell Medicine, New York, NY, USA.

Medical extended reality (MXR) systems, encompassing virtual reality (VR), augmented reality (AR), and mixed reality (MR), have emerged as transformative tools in medical education, offering immersive experiences by blending virtual environments with the real world.^1–3^ While the literature has extensively explored the applications of MXR in various surgical specialties, its utilization within anesthesiology and other more medically-oriented specialties remains under-examined.^4,5^ The scarcity of studies investigating the attitudes of anesthesiology residents towards MXR stands in stark contrast to the robust body of research within surgical training, highlighting an unexplored terrain in the realm of resident education. MXR may be particularly beneficial for anesthesia training as certain procedures, while seemingly routine, may be critical as lifesaving procedures performed under duress or care that improves patient outcomes in the perioperative setting, including endotracheal intubation, placement of central and arterial lines, and regional anesthesia. Simulation-based training using high fidelity mannequin simulators in anesthesiology training has been demonstrated to improve patient outcomes.^6^ However, high fidelity simulation training is expensive with direct costs encompassing the physical simulation laboratory and the mannequins themselves as well as indirect costs encompassing the need for clinical coverage for residents and faculty during simulation sessions. MXR enables increased access to simulation-based anesthesiology training, where procedures can be practiced asynchronously at any physical location whether at home or in the hospital. Thus, MXR is likely the more efficient and effective means for scaling simulation education in anesthesiology. Advancements in MXR technology also have showcased its potential in medical diagnostics through precise 3D radiographic imaging, providing an avenue for enhancing diagnostic skills and fostering a deeper understanding of complex anatomical structures.^1,3,4^ Additionally, MXR systems enable trainees to engage in realistic 3D simulations, offering a platform for practicing intricate operative techniques and conducting perioperative emergency simulations.^5^

Despite the progress in MXR technology, there remains a significant gap in understanding whether these advancements truly enhance perioperative education. Furthermore, the dearth of information regarding resident perceptions of MXR in anesthesiology training adds complexity to the broader conversation on the utility of MXR in medical education and what educational gaps, if any, trainees believe MXR may be most helpful in solving.

Our residency program within the Department of Anesthesiology recently hosted an introductory session on MXR for our 82-person residency as part of our “Mixed Teaching” curriculum that allows for exploration of health care systems topics, including technology and medical education. “Mixed Teaching” sessions comprise residents at all skills levels and various degrees of knowledge, skills, and attitudes. This session on MXR provided anesthesiology residents the opportunity to appreciate the scope of MXR based on the outline created by Brennan et al., demo use cases in both VR and AR with commercially available head-mounted devices providing a 3D experience of a digital environment, and conduct a post-session focus group with participants over the course of 60 minutes.^1^ An outline of the lesson plan for this session is presented in Figure 1. Further, there was an additional opportunity to engage residents with presession survey materials using online survey tools. An IRB was not required for this study since it constituted a category one exemption (integration within existing curriculum).

**FIG. 1.**
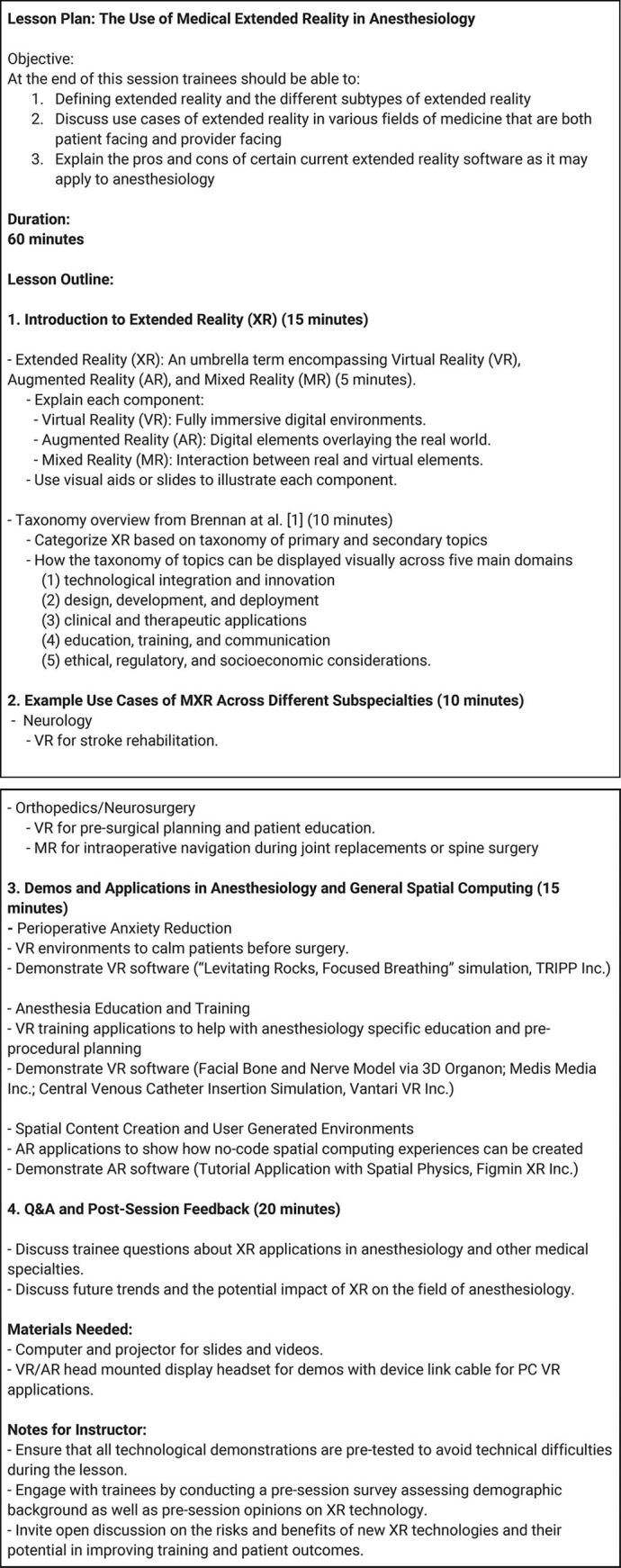
Description of the MXR Lesson Plan. MXR, medical extended reality.

There were 28 residents who participated in both the presession survey and the postsession focus group as a means to explore attitudes and perceptions of MXR within a single anesthesiology residency. Residents comprised all trainee levels within the anesthesiology residency and similar genders (Table 1).

**Table 1.** Participant Demographics

	N (%)
Age (years)	
20–25	0 (0%)
25–30	21 (75%)
30–35	7 (25%)
35–40	0 (0%)
Gender	
Male	12 (43%)
Female	16 (57%)
Year of Residency	
PGY1	5 (18%)
PGY2	9 (32%)
PGY3	7 (25%)
PGY4	7 (25%)
Previously used XR?	
Yes	20 (71%)
No	8 (29%)

Of the 28 participants in the session, the majority (79%) demonstrated a three or greater level of familiarity with XR on a 1–5 Likert scale (mean 3.14 [95% CI: 2.75, 3.53]), with 71% of participants indicating they have tried a form of XR at least once (Table 1). Those who have used XR previously used it for various purposes, including gaming, education, entertainment/social media, and clinical treatment. XR applications specifically in medical education, however, were less known, with 82% of respondents reporting low to moderate familiarity with how XR is used in medical education at the undergraduate and graduate level. Although there was decreased familiarity with XR in medical education, 100% of residents agreed there is a moderate to urgent need to improve anesthesiology training through technology on a 1–5 Likert scale (mean 3.57 [95% CI: 3.32, 3.82]). The majority (93%) expressed optimism regarding the potential of XR to improve resident education, with a mean of 8.03 (95% CI: 7.43, 8.63) on Likert 1–10 scale, specifically identifying procedural learning (96%), anatomy education (96%), and simulation training (82%) as areas that XR can improve. Furthermore, residents cited the opportunity for XR to provide an environment to explore generic or patient-specific anatomy and to practice procedures like regional blocks and arterial line placements. Interestingly, 71% respondents did not see XR as beneficial for soft skills and communication learning. Images from the trainee point-of-view during the session demos have been highlighted in Figure 2.

**FIG. 2.**
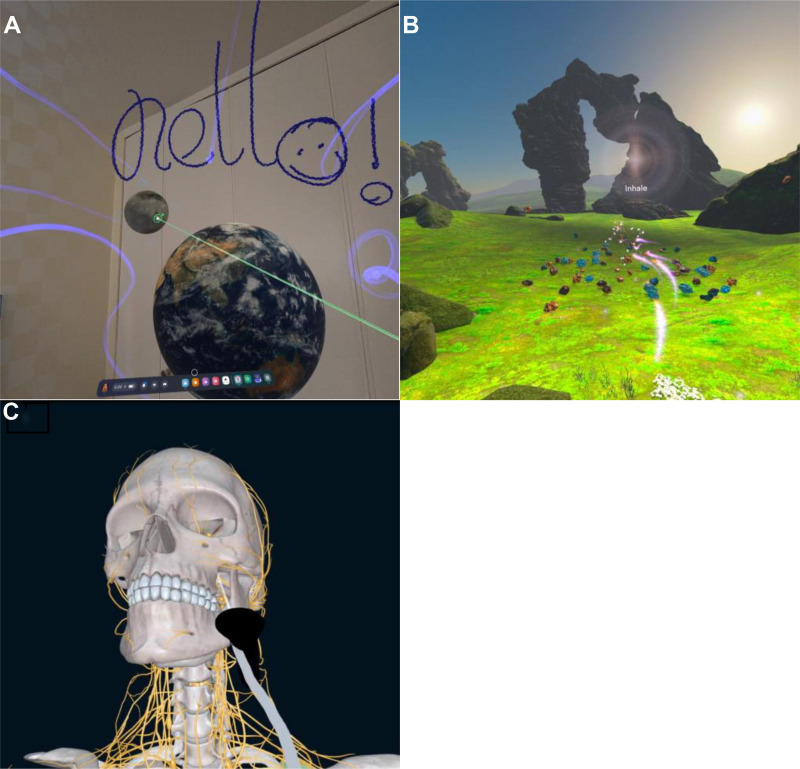
Screen Shots from Various Demos During the MXR Lesson.

Learner feedback from the focus group following the lecture confirmed the overall positive association the participants had with the potential for XR to improve residency education. The majority of residents echoed strong feedback stating they conceptually preferred lessons taught by a human teacher in shared virtual environments over autonomous learning in a gamified platform. This feedback potentially highlights perceived value from social interaction or from the teacher-learner relationship translated to immersive spaces. More research is needed to better understand the benefits of one pedagogical approach versus the other.

The study findings highlight a widespread familiarity with XR among respondents at one residency program, particularly in nonmedical contexts, yet a notable lack of awareness of its applications in medical education. Despite the use of MXR for medical education gaining popularity, there remains little published on MXR in anesthesiology, a specialty in which high-stake procedures are routine and simulation-based training is already documented to improve patient safety outcomes and embedded in the traditional training curriculum.^6^ Repetition with the same procedures is valuable, but barriers currently include access to the simulation laboratory and not facing critical situations like “difficult airways” in routine clinical settings. This likely explains why there is agreement among anesthesiology residents regarding the need to enhance training through technology and optimism surrounding XR’s potential to make training more accessible. The data suggest that current trainees have a very positive perception of XR’s role and potential in anesthesiology education and would potentially utilize these new learning modalities when available.

## Author Disclosure Statement:

RJ is Editor-in-Chief of the Journal of Medical Extended Reality and receives payments from Mary Ann Liebert, Inc. RJ is also a scientific advisor to 3D Organon, but receives no financial remuneration for his services. DC, AB, and JR have no conflicts of interests to disclose.

## Funding Information:

No funding was utilized for this research paper.

## Abbreviations Used

3D: Three Dimensional
AR: Augmented Reality
CI: Confidence Interval
IRB: Institutional Review Board
MR: Mixed Reality
MXR: Medical Extended Reality
VR: Virtual Reality
